# Determining blood flow direction from short neurovascular surgical microscope videos

**DOI:** 10.1049/htl.2019.0080

**Published:** 2019-11-26

**Authors:** Reid Vassallo, Adam Rankin, Stephen P. Lownie, Hitoshi Fukuda, Hidetoshi Kasuya, Benjamin W.Y. Lo, Terry Peters, Yiming Xiao

**Affiliations:** 1 Robarts Research Institute, Western University, London, Canada; 2 School of Biomedical Engineering, Western University, London, Canada; 3 Department of Clinical Neurological Sciences, Western University, London, Canada; 4 Department of Neurosurgery, Kochi University Hospital, Kochi, Japan; 5 Department of Neurosurgery, Tokyo Women's Medical University Medical Center East, Tokyo, Japan; 6 Department of Neurosurgery and Neurointensive Care, Lenox Hill Hospital, New York City, USA

**Keywords:** biomechanics, blood vessels, image segmentation, brain, bone, blood, augmented reality, biomedical optical imaging, biomedical ultrasonics, surgery, diseases, medical image processing, haemodynamics, neurophysiology, phantoms, blood flow direction, short neurovascular surgical microscope videos, neurovascular surgery, damaged blood vessels, static pre-operative images, routinely used surgical microscopes, blood pulsation, surgical videos, video segments, subtle colour fluctuations, Fourier space, physical phantom

## Abstract

Neurovascular surgery aims to repair diseased or damaged blood vessels in the brain or spine. There are numerous procedures that fall under this category, and in all of them, the direction of blood flow through these vessels is crucial information. Current methods to determine this information intraoperatively include static pre-operative images combined with augmented reality, Doppler ultrasound, and injectable fluorescent dyes. Each of these systems has inherent limitations. This study includes the proposal and preliminary validation of a technique to identify the direction of blood flow through vessels using only video segments of a few seconds acquired from routinely used surgical microscopes. The video is enhanced to reveal subtle colour fluctuations related to blood pulsation, and these rhythmic signals are further analysed in Fourier space to reveal the direction of blood flow. The proposed method was validated using a novel physical phantom and retrospective analysis of surgical videos and demonstrated high accuracy in identifying the direction of blood flow.

## 1 Introduction

Neurovascular surgery involves the repair of diseased blood vessels associated with the brain and spine. Overall, these procedures have a complication rate of 30.9% [1]. There are numerous types of surgeries that fall into this category, including arteriovenous malformation (AVM) resection, aneurysm repair, and carotid endarterectomy (CEA). Surgical microscopes are routinely used across all forms of neurosurgery [2], including neurovascular surgery.

AVMs are an abnormal collection of blood vessels which occur in the brain. Here, blood flows directly from an artery to a vein through a fistula [3]. AVMs have a reported prevalence of up to 0.5% [4], and may lead to seizures [5] and haemorrhage [6], potentially resulting in disability and death. The gold standard treatment for AVM is microsurgical resection [5].

During surgery, it is crucial to identify the direction of blood flow through the diseased region before resecting the AVM as clipping the wrong branch can result in severe complications. However, it is almost impossible to differentiate feeding from draining vessels in the AVM visually [7]. This is likely a contributing factor to 7.4% of AVM surgeries having complications that lead to permanent neurological deficits or death [8].

Aneurysms are another neurovascular disorder treated with surgery. An aneurysm is the ballooning of a weakened blood vessel wall and occurs in ∼2% of the population [9]. If left untreated, the aneurysm may rupture which can lead to stroke and possibly death. One treatment option to avoid a rupture is surgery involving clipping the aneurysm through a craniotomy.

Third, CEA is the gold standard treatment for carotid artery stenosis [10] to avoid the future risk of stroke. In this procedure, plaque is removed directly from the carotid artery. The surgeon will access the diseased carotid artery through an incision in the patient's neck, open the carotid artery, and remove the plaque.

Currently, blood flow can be highlighted by injecting fluorescent dye observable through surgical microscopes, known as the fluorescence videoangiography. However, this fails to provide continuous hemodynamic updates, as the dye is only visible for a short duration (∼10–15 s) after injection. After this, it cannot be reintroduced for >15 min as it remains in the patient's system. It also further complicates surgical workflows by requiring an injection and can have negative effects on patients' health, as there have been reported cases of adverse reactions to the dye [11–13].

Alternative solutions have been proposed, including the use of Doppler ultrasound imaging [14], augmented reality (AR) displays of preoperative images [15] and characterising periodic signals in intraoperative videos [13]. However, all these solutions have inherent limitations. The Doppler ultrasound approach risks rupturing fragile vessels by putting pressure on the already-weakened vessel wall. Ultrasound imaging also requires direct contact between the probe and the patient, which in this case may lead to relative movement of the brain tissue, a phenomenon known as brain shift. This means that the targeted vessels may no longer be where the clinician was planning on imaging them. The AR display approach can suffer from inaccurate real-time vessel identification [15]. Also, to keep the images in their proper location in the surgical scene, meticulous object tracking and camera calibration are required. This can complicate the surgical workflow and bring extra hardware into the operating room. The video analysis approach aims to identify vessels as feeders and drainers through blood pulsation strength but does not directly provide direction information.

Video enhancement methods [16, 17] that can reveal subtle hemodynamic differences in blood flow that are invisible to the naked eye have been applied to endoscopic surgery [18] and to study perfusion in the brain [19]. We aim to take advantage of the information that these methods provide and employ novel signal processing to detect the direction of blood flow in a vessel under investigation. The technique is particularly beneficial for AVM surgery, where there is the most ambiguity regarding the direction of blood flow. However, the resulting flow directions will also be useful for other vascular interventions. Therefore, in addition to validating our method using a novel vascular phantom, we also demonstrate the results with videos from AVM microsurgical resection, aneurysm repair, and CEA cases.

## 2 Methods

In this paper, we use video enhancement techniques to reveal subtle dynamic colour changes in videos. The phase information embedded in these enhanced changes is then further analysed to identify the direction of blood flow. All video processing and analysis were performed using MATLAB 2018b (The Mathworks, Natick, USA). We validated the method using a physical phantom, which simulates blood flow within a blood vessel segment, as well as with retrospective surgical videos.

### 2.1 Video enhancement

Our method relies on uncovering very subtle hemodynamic patterns. To do this, we used two video enhancement methods, found in [16, 17] as pre-processing steps in a similar manner to [13]. In general terms, these methods enhance dynamic information in three steps. First, the video frames are decomposed into a range of spatial frequency components that encode underlying information (such as motion or colour changes) at different rates. Then, the user-specified target frequency range is multiplied by the desired factor and will either be magnified or attenuated. Finally, the video is reconstructed by recombining these components.

Between these two methods, Eulerian video enhancement [17] is typically better at enhancing dynamic colour changes, while the phase-based approach [16] is better at motion magnification [20]. These algorithms have been implemented in real-time [18].

In this study, the videos were first stabilised using the algorithm described and implemented in [21] to address any large camera or object motions, which are modelled by an affine transformation using differential motion estimation. Then, the video underwent further motion attenuation using the phase-based approach to account for more deformable physiological motions, such as those induced by breathing [16]. Lastly, the colour changes from blood flow were amplified using the Eulerian video signal magnification [17], where the amplified colour changes of a frame $I_t$ are calculated with respect to a reference frame, ***I*** through

(1) $${I}^{\prime}_t\lpar x\comma \; y\comma \; c\rpar = I_t\lpar x\comma \; y\comma \; c\rpar + \alpha \delta _t\lpar x\comma \; y\comma \; c\rpar \eqno\lpar 1\rpar $$

where ${I}^{\prime}_t$ is the colour-amplified video frame, *x* and *y* are pixel coordinates, *c* is the channel of the colour image, $\alpha $ is the magnification factor and

(2) $$\delta _t\lpar x\comma \; y\comma \; c\rpar = I_t\lpar x\comma \; y\comma \; c\rpar - {\bi I}\lpar x\comma \; y\comma \; c\rpar \eqno\lpar 2\rpar $$

The selected frequency range was 0.8–3 Hz to ensure signals are detected even if the patient's intraoperative heart rate were to fall outside of the normal range of ∼60–100 bpm (1–1.67 Hz). Irregular vascular conditions may introduce abnormal blood pulsation rhythms.

### 2.2 Signal processing

To analyse the signals from the enhanced video, the green channel was used exclusively, as it provides the highest sensitivity to haemodynamics. This is because oxyhemoglobin has an absorption peak in the green range of the visible spectrum [22].

**Fig. 1:**
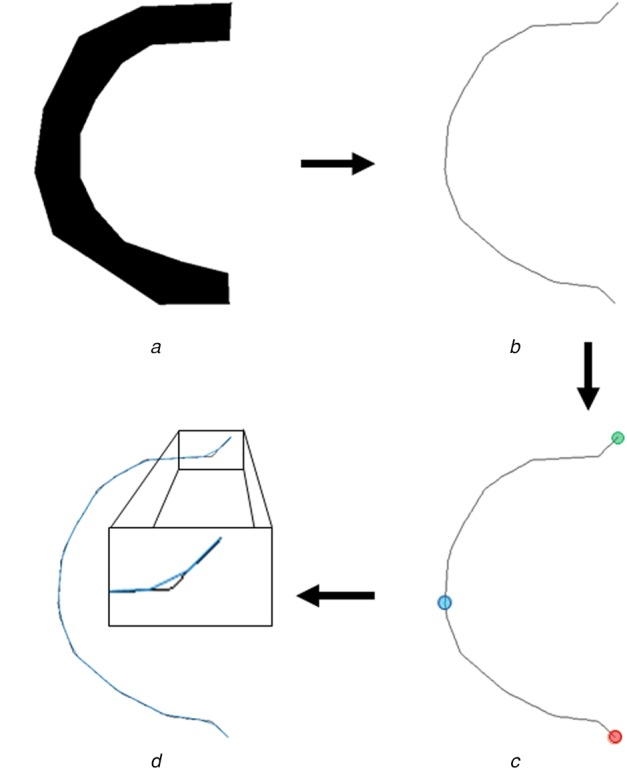
Overview of the steps taken to convert a generic labelmap into a polyline object. The steps progress clockwise, starting at the top-left. The Boolean images have been inverted for better visualisation *a* Generic labelmap for a vessel segment *b* Structural skeleton of the labelmap in *(a)*, *c* Same skeleton as *(b*) but three specific points are outlined. In blue is the first point to be queried in the process. In red is the final point found in the first round through the process, meaning it is one of the ends of this skeleton. The algorithm will repeat in the opposite direction, to include the entire skeleton, ending at the green point *d* Polyline object is downsampled by a factor of 25 and overlaid, in blue, on the original structural skeleton, in white

In order to automatically find the centreline of targeted vessels, we developed the following workflow. First, vessels are manually segmented with a polygon tool in MATLAB from the initial video frame. Next, a four-stage algorithm converts the segmentation labelmap to a polyline representing its centreline. This process is illustrated in Fig. 1 and described below.

The first stage uses MATLAB's skeletonisation function to automatically locate the structural skeleton of the segmented labelmap. The structural skeleton is converted to a polyline object using a four-stage workflow.

(i) The first linear index corresponding to a non-zero element is found as our initial point, and its coordinates are recorded, shown as the blue circle in Fig. 1*c*.
(ii) The next pixel is found which satisfies three conditions: it is a part of the skeleton, its coordinates have not previously been recorded, and it is connected to the current pixel. If a pixel satisfying these conditions are found, its coordinates are recorded and the process is repeated. If no such pixel is found, the end of the skeleton has been reached, shown as the red circle in Fig. 1*c*.
(iii) The recorded coordinates are cleared and this is repeated in reverse order. This approach guarantees a complete traversal of the skeleton regardless of starting conditions.
(iv) The list of points generated by the previous step is converted to a polyline and downsampled using the MatGeom toolbox (https://github.com/mattools/matGeom).

The amount of downsampling needs to consider a combination of the magnification, spatial resolution, and frame rate of the video under investigation and is automatically determined based on the amount of reliable information available. In all cases investigated in our study, the downsampling rate was empirically initialised at 25 (see the blue overlay in Fig. 1*d*). This was adequate for all clinical cases here. This results in a vertex spacing of ∼2 mm. Next, each frame is averaged separately such that each vertex receives the average green-channel value of all pixels to which it is the nearest-neighbour vertex.

**Fig. 2:**
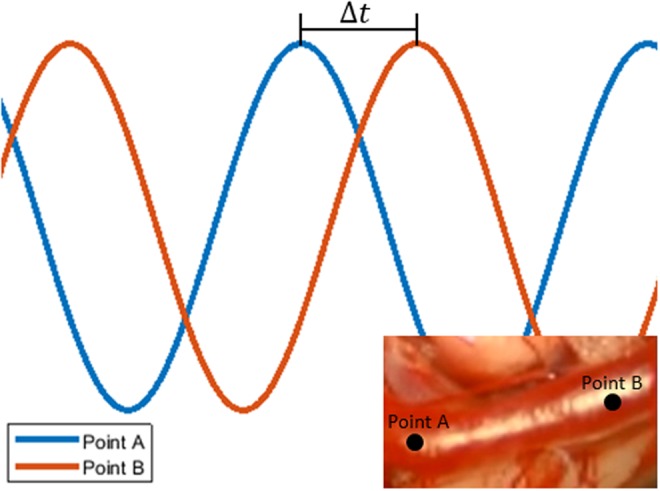
Example to demonstrate temporal offset $\left({\Delta t} \right)$ of blood pulsation signals at two points along a vessel. The points are marked on the vessel in the insert, and blood is flowing from Point A to Point B

When looking at the magnified colour fluctuations at a patch location of the blood vessels in the video, blood pulsation produces oscillating waveforms, which propagate along the direction of the blood flow. As a demonstration, ideal signals from two points along a vessel are shown in Fig. 2. The temporal offset of these two waveforms, denoted by $\Delta t$, is related to the phase difference in the Fourier domain through

(3) $$\Delta \theta = - 2\pi f\Delta t\eqno\lpar 3\rpar $$

where $\Delta \theta $ is the difference in phase between two signals, *f* is an arbitrary frequency, which will be heart rate in this application, and $\Delta t$ is the time delay. By detecting this phase difference in signals of blood flow, we can determine the average time differences between when a pulse of blood reaches two points along a vessel over several cycles.

A temporal Fourier transform is then taken of the video's average signals. The frequency component with the highest power is estimated to be the heart rate. The phase for this frequency component at every second polyline vertex is calculated, resulting in a value of $\theta $ such that

(4) $$Z = \vert Z\vert {\rm e}^{\,j\theta }\eqno\lpar 4\rpar $$

where *Z* is the video signal at the vertex, *j* is the imaginary unit, and $\theta $ is the phase in radians, in the range of [−$\pi $, $\pi $]. With the definition of phase angle in (4), $\theta $ will decrease when there is a positive time delay (i.e. as blood traverses along the vessel). Therefore, between two adjacent vertices, the direction of blood flow is deemed to go from the higher to lower value.

**Fig. 3:**
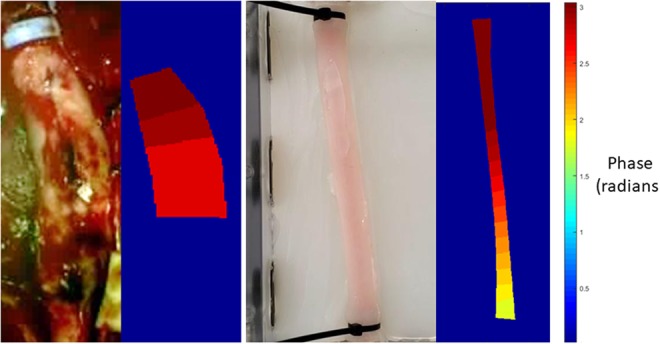
Representative images of the phase varying along the length of a vessel. The left images are frames from the video used to create the phase map (on the right). An example from a clinical case (left), an example video from the physical phantom (right)

This step can be visualised as a colourmap of phase values in the segmented vessel region, as seen in Fig. 3.

To ensure robustness, some steps are required, however, to reject detected phase shifts that are too small and likely due to noise, and those that are too large and likely due to phase wrapping. Phase wrapping, in this case, occurs when phase jumps from −$\pi $ to $\pi $ and will be explained more thoroughly later. To ensure that the phase shift is large enough, we propose that the value between two points must be at least above the detectable signal resolution by the optical device. More specifically, using heart rate as the frequency in (3), the phase shift of one pulsation cycle is obtained with

(5) $$\Delta \theta = - 2\pi f_{{\rm heart}}\Delta t\eqno\lpar 5\rpar $$

where $f_{{\rm heart}}$ is the patient's heart rate, in hertz. In this method, $f_{{\rm heart}}$ is determined as the component of the frequency spectrum of the video's average signal with the highest magnitude. It is assumed that blood flow due to heart activity is the only major source of signal oscillation. For a given time, using a camera with a fixed frame rate, the minimum phase shift that can be reliably detected is:

(6) $$\vert \Delta \theta \vert \ge \displaystyle{{2\pi f_{{\rm heart}}} \over {\,f_{{\rm video}}}}\eqno\lpar 6\rpar $$

where $\Delta \theta $ is once again the difference in phase between two points and $f_{{\rm video}}$ is the video frame rate in Hz.

Additionally, since values of phase are constrained to the range $\lsqb - \pi \comma \; \pi \rsqb $, it is possible that as $\theta $ decreases, it could seem to jump from $ - \pi $ to $\pi $ and then continue to decrease due to convention in digital processing. This is known as phase wrapping, and here it artificially goes against the expectation that $\theta $ will decrease along the direction of blood flow. This will cause a phase difference of ∼$2\pi $. More realistically, to reject values of $\Delta \theta $ that are too large, we require

(7) $$\vert \Delta \theta \vert \le \pi \eqno\lpar 7\rpar $$

where $\Delta \theta $ is once again the difference in phase between two points.

Thus, combining (6) and (7) we obtain the condition required to accept a value of $\Delta \theta $ as a reliable measure

(8) $$\displaystyle{{2\pi f_{{\rm heart}}} \over {\,f_{{\rm video}}}} \le \vert \Delta \theta \vert \le \pi \eqno\lpar 8\rpar $$

### 2.3 Automatic video annotation

To intuitively display the processed information, an automatically annotated display is required. As the direction of blood flow in a given vessel is not expected to change through the duration of a procedure, the information is overlaid on a representative frame from the surgical video.

An arrow in a high-contrast colour is automatically overlaid onto the surgical video frame using MATLAB. These arrows span vertices of the aforementioned polyline, where the phase differences between vertices obey (8).

### 2.4 Validation

The validation of this technique was done using a physical phantom and retrospective clinical video analysis.

#### 2.4.1 Phantom study

A physical vessel phantom was created for this study by indirect three-dimensional (3D) printing, similar to the method described in [23]. The exterior and interior (lumen) geometries of a simplified vessel, and their fixture points to an external container, were designed using the Computer-Aided Design (CAD) software SpaceClaim (ANSYS Inc., Canonsburg, USA) and 3D printed using an Ultimaker 3 Extended printer (Ultimaker BV, Geldermalsen, The Netherlands).

First, the exterior shape was rigidly affixed to the walls of the container, which was filled with Ecoflex 00-30 silicone (Smooth-On, Macungie, USA) in two steps. Once the silicone had cured and the 3D printed parts were removed, this created a negative mould. Next, the interior geometry was inserted inside the mould, which was injected with Ecoflex 00-30 silicone. The final product was simplified vessel geometry with 3 mm thick walls.

**Fig. 4:**
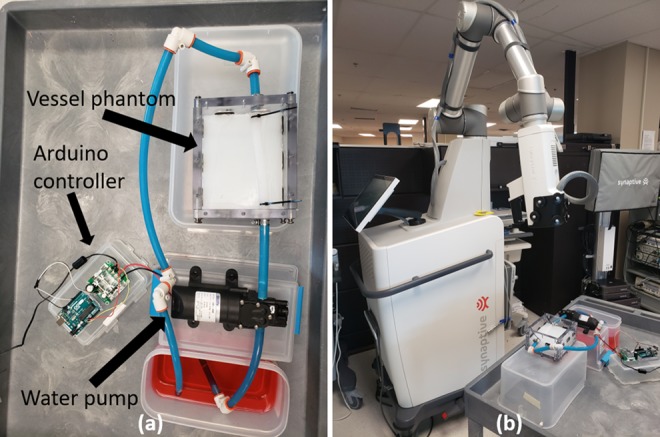
Views of the physical phantom validation experiments *a* Physical vessel phantom, water pump, Arduino controller and dyed water *b* Capturing video using the Synaptive Modus V surgical exoscope

This phantom was attached to a water pump, which was controlled with an Arduino microcontroller (www.arduino.cc) to mimic a cardiac cycle. Specifically, it was periodic with a primary period of 1 s, where it was on for $1/3$ seconds and off for $2/3$ seconds to approximately simulate the systolic and diastolic time fractions of the cardiac cycle [24]. The water in this system was dyed red to add contrast. The setup of this phantom can be seen in Fig. 4*a*

The video was recorded using a Modus V surgical exoscope (Synaptive Medical, Toronto, Canada) at 60 frames per second (fps). This setup can be seen in Fig. 4*b*.

#### 2.4.2 Retrospective video analysis

Following validation using a physical phantom, videos of surgical cases were analysed retrospectively. Upon informed consent, short videos were retrospectively obtained from microscopes used during neurovascular surgeries. These cases included one AVM resection, one aneurysm repair, and two CEAs from different institutions. In each instance, a short video segment (5–10 s) of the exposed surgical site, which was free from motion of the camera or surgical tools, was taken for processing and analysis. In these cases, the operating surgeon confirmed the direction of blood flow in the vessel to create our ground truth. In the AVM and aneurysm cases, this ground truth was determined using the standard of care at that institution with the help of pre-operative imaging data. The surgeries were successful, so it can be reasonably expected that the ground truth is correct. In the CEA cases, the ground truth blood flow direction is known from basic anatomy. These videos all have a frame rate of 30 fps.

## 3 Results

These results of this study have been split between the phantom validation and retrospective video analysis.

### 3.1 Phantom study

**Fig. 5:**
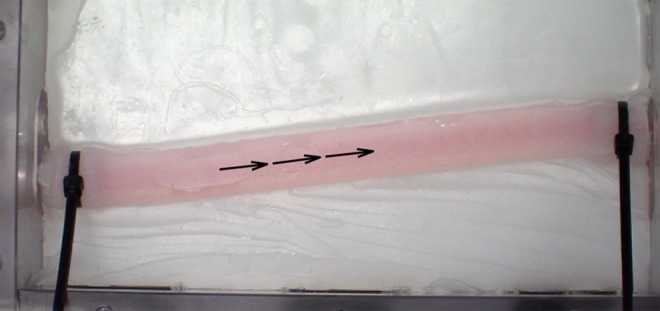
Results of this algorithm being applied to the video of our vessel phantom, which has correctly identified the direction of blood flow

The correct blood flow direction was determined in the phantom video. The resulting annotated view is shown in Fig. 5. The signal processing and automatic annotation of this video took 182.14 s.

### 3.2 Retrospective video analysis

**Fig. 6:**
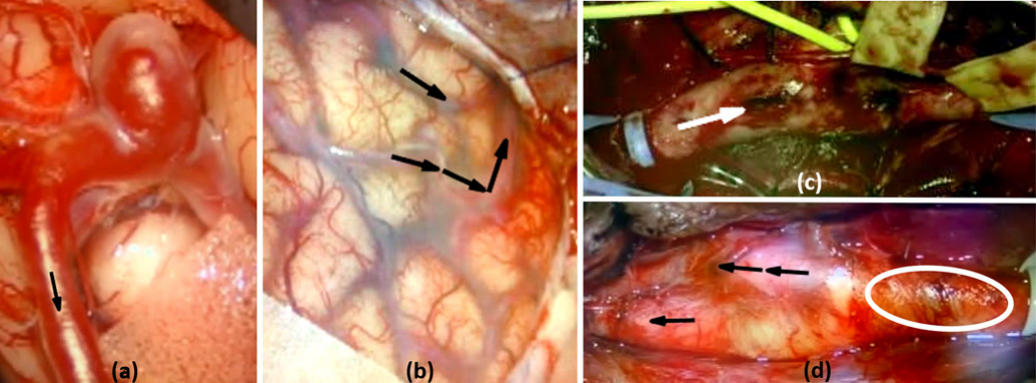
AR views of the four videos retrospectively evaluated in this study *a* The resulting AR view from an aneurysm case, correctly identifying the blood flow direction *b* The resulting AR view from an AVM resection case. Both segments here correctly identify the blood flow direction *c* The AR view from one CEA case. One vessel segment was investigated here, and the blood flow direction was correctly identified *d* The AR view from a second CEA case. Three segments of this vessel were investigated, two resulting in the correct estimations and a third (white circle) resulting in no reliable information to estimate blood flow direction

**Fig. 7:**
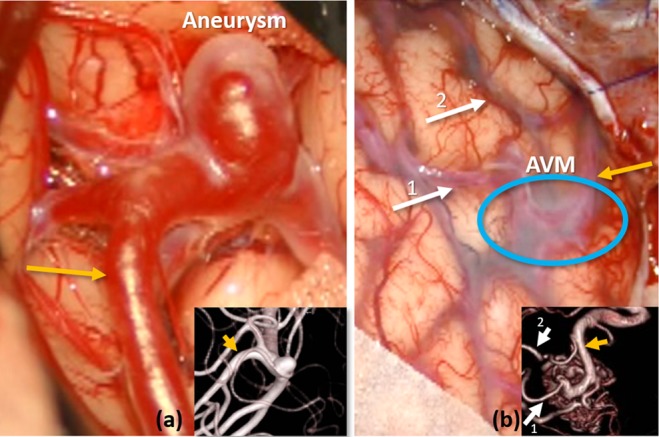
Annotated views of the surgical videos of the aneurysm and AVM cases, including pre-operative angiography (inserts) *a* An annotated view of the ground truth of the aneurysm case. The draining vessel is labelled with a yellow arrow *b* An annotated view of the ground truth of the AVM case. The feeding vessels are labelled with a white arrow, and the draining vessel with a yellow arrow. The location of the AVM is circled in blue, but the AVM is below the surface

The annotated views of results from all four cases are displayed in Fig. 6. The correct flow direction is identified in six of seven vessel segments investigated. One segment (white circle) did not provide any information that satisfied (8), so no direction could be estimated reliably. For this CEA case, the relevant segment contained a plaque to be removed. Thus, incorrect flow information was not provided in any of these preliminary results. For context, the pre-operative angiography and other annotations of the AVM case are included in Fig. 7. The signal processing and annotation of these videos took an average of 7.63 $ \pm $ 0.53 s.

## 4 Discussion

This work demonstrates the preliminary validation of a method to estimate the direction of blood flow in vessels solely though videos acquired from routinely used surgical microscopes. A high degree of accuracy has been demonstrated, as all estimates based on reliable information are correct. This is demonstrated by the gaps in arrows in Fig. 5, as well as the encircled area in Fig. 6*d*. This method was validated using videos taken at different institutions with different surgical microscopes, further confirming the robustness of the proposed technique.

The proposed method overcomes the limitations of previously proposed solutions to estimate blood flow intraoperatively [14, 15]. Specifically, it does not require any contact with the patient's brain or any contrast agents, and uses real-time hemodynamic information that can be continuously updated as the procedure progresses.

To demonstrate this method, first, a novel vessel phantom was created by an indirect 3D printing process. This was attached to a pump which was controlled to mimic the cardiac cycle and used to validate this method on video from a clinical-grade surgical microscope. This method was also validated retrospectively on a series of clinical videos. With the ability to easily change the design, we expect the introduced vessel phantom construction to be useful for wider applications as well. The phantom study took much longer to run than the clinical videos because the video had a much larger frame size and more frames due to its higher frame rate.

This method works best under the assumption that there are no moving surgical tools in the scene or major patient motion. As only 5–10 s of video is required, this is a reasonable expectation in surgical procedures. Also, steps have been put in place to correct for what small movements may still be present in the video.

Integrating this with clinical systems capable of higher frame rates would be advantageous. Higher frame rates allow for a finer resolution of phase differences, which is particularly useful in clinical cases with only a short segment of vessel visible. Also, clinical integration would allow for contactless determination of blood flow direction without any contrast agent, saving time and eliminating a potential source of complications. This can help make these procedures safer and more effective.

### 4.1 Future work

Immediate future work of this project includes more robust implementation with the video enhancement and analysis algorithms. Recently, methods based on dynamic linear modelling have demonstrated more accurate predictions of heart rate and perfusion than Fourier-based methods [25], and may be beneficial for our implementation. The current technique is implemented using MATLAB, and the processing speed can be further enhanced with C + + and parallel programming.

In order to fully automate this method, gathering more cases will allow us to begin leveraging deep learning to automatically segment the vessels from the video [26]. However, the manual segmentation, as is used here, is robust and with the right segmentation software, a manual segmentation can be acquired with only a few strokes [27].

As part of our future work, the introduced method will be more rigorously validated on additional clinical cases, as we are actively collecting surgical data. This includes cases with moving surgical tools in the scene to better understand their impact on the relevant technique and to devise more adaptable solutions. We also plan to adapt the phantom design and setup to include more varied conditions so we may obtain an even better characterisation of our method. Finally, this method will also be compared to other techniques that can intraoperatively determine blood flow direction such as [14] regarding accuracy and ease of use.

## 5 Conclusion

This paper proposes a new video-based technique to identify the direction of blood flow in vessels. This has been validated using a physical phantom as well as a set of retrospective surgical videos. By magnifying subtle colour changes and isolating those which belong to the heart rate frequency, we are able to track these changes through time to identify the direction of blood flow with good accuracy.
